# Bronchoalveoloar lavage fluid cytokines and chemokines as markers and predictors for the outcome of interstitial lung disease in systemic sclerosis patients

**DOI:** 10.1186/ar2766

**Published:** 2009-07-17

**Authors:** Katrin Schmidt, Lorena Martinez-Gamboa, Susan Meier, Christian Witt, Christian Meisel, Leif G Hanitsch, Mike O Becker, Doerte Huscher, Gerd R Burmester, Gabriela Riemekasten

**Affiliations:** 1 Department of Rheumatology and Clinical Immunology, Charité-Universitätsmedizin Berlin, Charitéplatz 1, 10117 Berlin, Germany; 2 Department of Radiology, Charité-Universitätsmedizin Berlin, Charitéplatz 1, 10117 Berlin, Germany; 3 Department of Infectiology and Pulmonology, Charité-Universitätsmedizin Berlin, Charitéplatz 1, 10117 Berlin, Germany; 4 Institute of Medical Immunology, Charité-Universitätsmedizin Berlin, Charitéplatz 1, 10117 Berlin, Germany; 5 German Rheumatism Research Centre, Charitéplatz 1, 10117 Berlin, Germany

## Abstract

**Introduction:**

Interstitial lung disease (ILD) is a frequent manifestation of systemic sclerosis (SSc), and cytokines can contribute to the disease pathology. The aim of the current study was to identify specific changes in cytokine levels that may serve as disease markers and possible targets for therapy.

**Methods:**

Cytokines were measured with bioplex analysis in 38 bronchoalveolar fluids (BALFs) from 32 SSc patients (27 with alveolitis and 11 without alveolitis) and 26 control patients. In the case of SSc patients, cytokines were correlated with the respective bronchoalveolar lavage (BAL) cell differentiation, lung function, and thoracic HR-CT score. For 35 BALF samples derived from 29 SSc patients, follow-up investigations of clinical data, lung-function parameter, or thoracic HR-CT scans were available to evaluate the predictive capacity of BALF cytokines and chemokines.

**Results:**

High IL-7 levels were characteristic of SSc-associated interstitial lung disease (ILD) and, in addition, when compared with ILD-negative SSc patients, ILD-positive SSc patients revealed higher IL-4, IL-6, IL-8, and CCL2 (MCP-1) BALF levels. High CCL2 and IL-8 BALF concentrations were associated with neutrophilic and mixed alveolitis. Cytokine levels of IL-4, IL-8, and CCL2 correlated negatively with lung-function parameters; CCL2 concentrations also correlated with HR-CT scores. High concentrations of several cytokines were associated with the progress of ILD and end-stage ILD. Univariate analyses revealed high IL-2 and tumor necrosis factor-alpha (TNF-α) levels as the best predictors for progressive disease, together with lung-function parameters, young age, and neutrophilic alveolitis. Multivariate analyses partially confirmed these results but did not sufficiently converge because of the limited number of patients.

**Conclusions:**

The association of BALF cytokines with lung fibrosis and its progress suggests that cytokines contribute to the pathogenesis of ILD and hence could be regarded as potential therapeutic targets.

## Introduction

Systemic sclerosis (SSc) is an autoimmune disease characterized by fibrosis of the skin and various internal organs. Interstitial lung disease (ILD) and its complications represent the most prominent causes of death in SSc. Alveolitis develops in up to 80% of SSc patients, and progression to end-stage fibrosis occurs in about 15% [1]. Unfortunately, factors that predict progression and poor prognosis are missing. Cellular differentiation of bronchoalveolar lavage (BAL) cells is often used to define alveolitis. In addition, neutrophilic alveolitis has been suggested to predict the progression of fibrosing alveolitis [2]. In a recent multicentric study including 141 patients, BAL neutrophilia was associated with early and overall mortality, but the effect on overall mortality was lost when disease severity was taken into account [3]. The authors concluded that BAL findings add only limited prognostic information in SSc-related interstitial lung disease in addition to HR-CT scans and lung-function parameters (LFP) [3,4]. Nevertheless, the authors argued that other markers might reflect disease progress and the pathogenic mechanisms present in SSc-ILD.

The role of chemokines and cytokines as markers reflecting disease severity and predicting outcome in SSc-related lung disease has not been studied extensively. Chemokines are important regulators of cell migration and the recruitment of leukocytes to specific tissue sites [5]. Among them, monocyte chemoattractant protein-1 (MCP-1 or CCL2) and macrophage inflammatory protein-1β (MIP1β or CCL4) may play a role in SSc, as the overexpression of these chemokines has been detected in SSc-related lung disease [6,7]. In addition to chemokines, cytokines such as IL-6 or TGF-β also can mediate different pathogenic processes in systemic sclerosis. Polymorphisms of several cytokines found to be associated with SSc and involved in the regulation of fibrosis support their role in SSc pathogenesis [8,9]. Therefore, both chemokines and cytokines could play a role in the pathogenesis of SSc-ILD and as targets of future therapies [10].

In the present investigation, we have determined levels of cytokines and chemokines in BAL fluids (BALF) in an early SSc cohort. Furthermore, we analyzed controls with ILD due to other diseases to identify key cytokines specifically involved in the pathogenesis of SSc-related lung disease. Furthermore, in a cross-sectional study, the correlation of cytokine and chemokine levels with signs of lung fibrosis was studied. Finally, by follow-up investigations of the clinical data, lung function, and HR-CT scores, the predictive value of cytokines and chemokines was evaluated. We have identified key cytokines that appear to be associated with lung fibrosis and that may predict worsening of ILD in SSc patients.

## Materials and methods

### Patients

The 38 bronchoalveolar lavage fluid (BALF) samples were obtained from 32 SSc patients and 26 patients with other diseases between 2004 and 2006. SSc patients (20 with diffuse and 12 with limited SSc) fulfilled the preliminary criteria for the disease classification of SSc [11]. Epidemiologic data of patients at the time of BAL are presented in Table 1. Mean prednisone doses in SSc patients and in control patients were 5 mg/d and 5.6 mg/d, respectively. In the control group, 20 patients had alveolitis, and among them, 12 had sarcoidosis, and six patients had idiopathic interstitial lung disease. One patient had broncheolitis obliterans and another, alveolar proteinosis. Six patients with normal BAL cell differentiation and no lung pathology were defined as healthy persons. BAL was carried out when indicated (to diagnose or exclude ILD, infections, or malignant diseases), with the written informed consent of patients for diagnostic or clinical purposes. Patients with present pulmonary infections were excluded from the study. The study was approved by the local ethics committee (EA1/013/705). Written consent was obtained from each patient.

**Table 1:** Demographic characteristics of the patients

	SSc (n = 32)	Sarcoidosis (n = 12)	Other ILD (n = 8)	Healthy donor (n = 6)
Age (years)	58.5 (30–72)	47 (30–67)	56.5 (24–78)	41 (20–57)
Female/male	23/9	8/4	4/4	4/2
Smoker/ex/NS	3/9/20	4/2/6	1/1/6	1/0/5
Recovery (percentage)	73.5 (47–90)	73.7 (60–80)	80 (50–83)	71.6 (53–77)
Neutrophils	3 (0–49)	2 (0–6)	15.5 (0–56)	3 (0–3)
Lymphocytes	8 (0–48)	27 (13–62)	20.5 (6–70)	6 (3–9)
Eosinophils	1 (0–15)	0 (0–1)	0.25 (0–3)	0 (0–0.5)
Macrophages	81.5 (32–99)	69 (31–82)	55 (24–82)	90 (88–94)

Median values and ranges (in parentheses) are shown.

### Assessment of the patients

For cross-sectional analyses, patients were assessed for signs of lung fibrosis with lung-function tests (LFTs) or with high-resolution computed tomography (HR-CT) scans, including HR-CT scores (Aquilion 16/Aquilion 64, Toshiba Medical Systems, Zoetermeer, The Netherlands). Furthermore, for the evaluation of fibrotic skin changes, the modified Rodnan Skin Score (mRSS) was used [12]. Pulmonary fibrosis was defined by evidence of fibrosis such as bibasilar fibrosis on chest radiograms or HR-CT scans or both. Spirometry and body plethysmography (Siregnost FD 40/FD 91, Siemens, Erlangen, Germany) were performed to determine forced vital capacity (FVC) and total lung capacity (TLC). The pulmonary diffusing capacity for carbon monoxide (DLCO) was determined with the single-breath method (DLCO-SB; Transferscreen II, Fa. Jäger, Würzburg, Germany). Values for TLC, FVC, and DLCO were expressed as percentages of predictive normal values adjusted for age, sex, and height. For the longitudinal study, follow-up of LFTs and HR-CT scores was performed at a mean period of 49 weeks and 58 weeks, respectively. Clinical data also were obtained for SSc patients. Deterioration of lung-function parameters (predicted FVC and DLCO-SB) was defined by changes of 10% or more. Progressive lung disease was defined by worsening of at least one lung function parameter by 10% or more or by an increase in HR-CT scores of 3 or more, or both. If the HR-CT scan was not available for scoring, progressive disease was defined by the consent of two experienced radiologists. In addition, end-stage ILD was defined either by death or by the need for continuous oxygen supplementation.

### CT scan and visual analysis

CT scans were performed by using a CT scanner (Aquilion 16/Aquilion 64) 3 or fewer months before BAL. Acquisition was done by using the 0.75-mm detectors; images were reconstructed in 0.5-mm slice widths. Thin-section CT scans of the lungs were independently evaluated by two radiologists independently on a GE Workstation at fixed window width of 1,500 Hounsfield units (HU) and level (-500 HU). Visual evaluation included a score of severity and a score of extent (range, 0 to 30) and was performed as described [13]. To assess intraoperator reproducibility, one radiologist (S.M.) repeated the visual assessment in all patients 3 times, separated by at least 24 hours.

### BAL procedure and recovery of BALF

BAL was performed as recommended by the American Thoracic Society according to the task-force guidelines and as described previously by using an Olympus BF 1T20 fiberoptic bronchoscope (Olympus Europe, Hamburg, Germany) [14,15]. In brief, the bronchoscope was wedged into a segment bronchus of the right middle lobe, and 150 ml of 0.9% sodium chloride solution (37°C) was instilled and gently aspirated. BAL differential cell counts were performed on cytospin preparations stained with the May-Grünwald-Giemsa method. According to normal values obtained by the same BAL procedure [16], the following BAL differential cell counts were classified as pathologic in nonsmokers: more than 15% lymphocytes, more than 3% neutrophils, more than 0.5% eosinophils, or a combination of these; in smokers, more than 7% lymphocytes, more than 3% neutrophils, more than 0.5% eosinophils, or a combination of these. Alveolitis/ILD was defined as an increase in the proportions or absolute numbers (or both) of inflammatory cells present in BAL fluid [17]. Pathologic BAL cell counts were differentiated into lymphocytic, neutrophilic, eosinophilic, and mixed forms (combination of lymphocytosis and granulocytosis).

### Bioplex analysis

Cytokine concentrations adjusted according to the recovery rate of BALFs were determined by using the Bio-Plex Protein Array System (Bio-Rad, Hercules, CA, USA). Cytokine-specific antibody-coated beads (Bio-Rad) were used for these experiments. The assay was performed according to the manufacturer's instructions. Cytokine concentrations were automatically calculated with Bio-Plex Manager software by using a standard curve derived from a recombinant cytokine standard. According to previous experiments analyzing 17 cytokines (IL-1, IL-2, IL-4, IL-5, IL-6, IL-7, IL-8, IL-10, IL-12, IL-13, IL-17, CCL2, CCL4, TNF-α, G-CSF, GM-CSF, and INF-γ) derived from BALF samples of 11 SSc patients as well as from 15 controls, the following cytokines were selected for further analyses of all BALF samples: IL-4, IL-6, IL-7, IL-8, IL-10, CCL2, CCL4, G-CSF, and TNF-α.

### Detection of TGF-β1 in BALF and sera

For the detection of TGF-β1 concentrations, a commercially available ELISA was used and performed according to the manufacturer's instructions (Quantikine Human TGF-β1, R&D Systems, Wiesbaden, Germany). The recommended dilution of sera (1:40) and of BALF (1:20) revealed values below the detection level. Therefore, sera and BALF were diluted 1:20 and 1:5, respectively. Values were corrected according to the dilution and BALF recovery.

### Statistics

GraphPad Prism Version 3.02 (GraphPad Software, San Diego, CA, USA) for Microsoft^®^, Windows, was used for statistical analysis. The nonparametric Mann-Whitney *U *test was performed to compare cytokine levels in different groups. *P *values lower than 0.05 were considered statistically significant. Linear correlation was estimated by the Pearson correlation coefficient. Logistic regression analysis was performed by using the SPSS V 15.0 statistical package. BALF cytokine concentrations were examined with univariate analysis, as well as age, gender, DLCO-SB, FVC, HR-CT score, mRSS, neutrophilic and eosinophilic alveolitis, and BALF cytokines. Multivariate analysis was performed with those parameters selected by univariate analyses with *P *values less than 0.1.

In a second multivariate analysis, only BALF cytokines were studied. Multiple samples from one patient were accordingly weighed for analysis. Based on the pilot character of the study in patients with a rare disease, *P *values were not adjusted for multiple testing.

## Results

### Patients with systemic sclerosis have specific cytokine changes

As shown in Table 2, SSc-associated alveolitis is characterized by specific BALF cytokine changes. In SSc patients with ILD, IL-7 concentrations were higher compared with those found in patients with ILD due to other diseases. When ILD in SSc patients was compared with the ILD due to sarcoidosis, higher IL-8 levels in addition to higher IL-7 levels were detected. BALF analyses of idiopathic ILD patients were characterized by lower IL-7 and IL-10 concentrations compared with those of SSc-ILD patients (Table 2).

**Table 2:** Median BALF cytokine concentrations and ranges in SSc patients with and without alveolitis compared to different controls

Cytokine	Median concentration in BALF from SSc patients (range)	Median concentration in BALF from control patients (range)	*P *values
SSc (n = 38) versus all controls (n = 26)			
IL-6	17.72 (1.7–177)	25.5 (6.3–567)	0.027
IL-7	4.43 (0–17.4)	1.95 (0–8.6)	0.0123
SSc alveolitis (n = 27) versus alveolitis due to other disease (n = 20)			
IL-7	4.88 (0.75–17.4)	1.95 (0–8.6)	0.0037
SSc alveolitis (n = 27) versus alveolitis due to sarcoidosis (n = 12)			
IL-7	4.88 (0.75–17.4)	2.01 (0–8.6)	0.0414
IL-8	105.5 (14.9–754)	46.3 (13.0–191.8)	0.0372
ILD-positive SSc (n = 27) versus idiopathic ILD (n = 6)			
IL-7	4.88 (0.75–17.4)	2.0 (0–4)	0.0423
IL-10	2.23 (1.3–6.7)	1.75 (0–2.1)	0.0297
IL-4	3.47 (0–22.9)	0 (0–5.2)	0.048
IL-6	20.9 (1.7–177)	13.4 (6.2–20.0)	0.0496
IL-7	4.88 (0.75–17.4)	2 (0–5.8)	0.0461
IL-8	106 (14.9–794)	47 (5.9–223)	0.0132
CCL2	92.2 (14.1–2001)	24.1 (0–97.5)	0.0018
ILD-positive SSc (n = 27) versus ILD-negative controls without any lung disease (n = 6)			
IL-7	4.88 (0.75–17.4)	2 (0–8.2)	0.014
IL-8	106 (14.9–794)	31 (0–112.2)	0.0143
CCL2	92.2 (14.1–2001)	31.6 (14.4–42.6)	0.0051
CCL4	46.0 (24.6–350)	21.6 (2.8–58.8)	0.0048
TNF-α	1.2 (0–8.1)	0 (0–0.6)	0.01

Concentrations are given in picograms per milliliter, with corresponding *P *values. Only cytokine concentrations with significant differences between the compared groups are shown.

When comparing ILD-positive SSc patients with ILD-negative SSc patients, IL-4, IL-6, IL-7, IL-8, and CCL2 levels were significantly increased in the ILD-positive SSc patients. Compared with ILD-negative healthy controls, ILD-positive SSc patients showed higher IL-7, IL-8, and CCL2 levels (Table 1). In addition, ILD-positive SSc patients revealed higher TNF-α and CCL4 levels. BALF TGF-β1 and IL-13 levels were below the detection level in SSc patients (data not shown). ILD-positive patients with other diseases revealed a different cytokine/chemokine pattern. In patients with idiopathic ILD, only increased CCL4 (median, 126.9 pg/ml) and CCL2 (132.2 pg/ml) concentrations were found compared with those in ILD-negative healthy controls (*P *= 0.0043 and *P *= 0.026; data not shown). ILD due to sarcoidosis was characterized by increased cytokine levels of TNF-α compared with healthy donors (1.2 versus 0 pg/ml). The other BALF cytokine levels did not show any significant differences.

### Cytokine levels of IL-8, CCL2, and IL-6 are highest in patients with neutrophilic alveolitis and are not secondary phenomena due to the BAL cellular constituents

**Figure 1:**
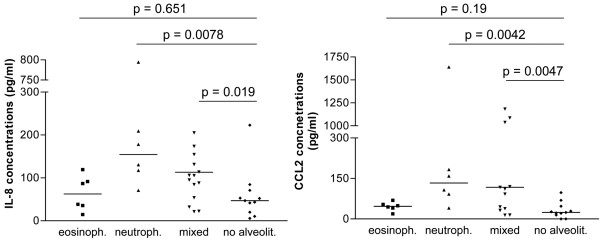
Bronchoalveolar lavage fluid (BALF) cytokine levels of interleukin 8 (IL-8) **(a)** and CCL2 **(b)** in systemic sclerosis (SSc) patients are associated with BAL cell differentiation and alveolitis subgroup. Cykokine levels found in BALF from patients with neutrophilic, mixed, and lymphocytic alveolitis were compared. SSc patients without any signs of alveolitis are used as controls.

Concentrations of only few cytokines were associated with the dominant BAL cellular constituent determining the type of alveolitis. IL-8 levels from SSc patients were high in patients with neutrophilic and mixed alveolitis (median, 250.3 and 105.5 pg/ml, respectively) compared with SSc patients with normal BAL cell values (47.0 pg/ml, Figure 1a). Patients with lymphocytic alveolitis did not show increased IL-8 levels. Similar results were found for the CCL2 levels (Figure 1b). Only patents with neutrophilic alveolitis revealed higher IL-6 concentrations compared with SSc controls (27.3 pg/ml versus 1.9 pg/ml; *P *= 0.002; data not shown). Mixed lymphocytic/neutrophilic alveolitis was characterized by increased IL-2 levels compared with ILD-negative SSc patients (*P *= 0.02, data not shown). To evaluate whether BALF cytokine concentrations are secondary phenomena due to the different cellular constituents, we correlated both the percentage and the absolute number of the different cell types with BALF cytokine concentrations in SSc patients and controls (Table 3). In SSc patients, other cytokine and chemokine concentrations correlated with the absolute number or percentages of cells compared with controls. Thus, IL-6 and CCL2 levels correlated with the percentage of eosinophils in controls, but not in patients with SSc. In general, more correlations between the percentages or absolute numbers of the cellular compounds were found in controls compared with the SSc patients (Table 3).

**Table 3:** Correlation between the percentage and absolute number of BAL cells per milliliter recovery and different BALF cytokines

	GCSF	IL-1β	IL-2	IL-4	IL-6
	
SSc					
Percentage of cells					
Eosinophils					
Lymphocytes					
Neutrophils	0.490^b^	0.369^a^	0.433^a^	0.544^b^	
Absolute number of cells per millilitre of recovery fluid					
Eosinophils					
Lymphocytes					
Neutrophils	0.672^c^	0.489^b^		0.599^c^	
Controls					
Eosinophils					
Controls					
Percentage of cells					
Eosinophils	0.647^c^	0.412^a^	0.541^b^	0.669^c^	0.575^b^
Lymphocytes					
Neutrophils		0.490^a^			0.512^b^
Absolute number of cells per millilitre of recovery fluid					
Eosinophils	0.532^b^	0.924^c^		0.585^b^	
Lymphocytes		0.627^b^			
Neutrophils		0.912^c^		0.544^b^	

	IL-8	IL-10	CCL2	CCL4	TNF-α
	
SSc					
Percentage of cells					

Eosinophils					
Lymphocytes				0.392^a^	
Neutrophils	0.604^c^		0.628^c^		0.622^c^
Absolute number of cells per millilitre of recovery fluid					
Eosinophils					
Lymphocytes					
Neutrophils					0.549^b^
Controls					
Percentage of cells					
Eosinophils	0.586^b^	0.592^b^	0.770^c^	0.526^b^	0.813^c^
Lymphocytes				0.594^b^	
Neutrophils			0.472^a^		0.453^a^
Absolute number of cells per millilitre of recovery fluid					
Eosinophils	0.608^c^			0.869^c^	
Lymphocytes	0.425^a^	-0.396^a^		0.772^c^	
Neutrophils	0.718^c^	0.536^b^		0.852^c^	0.647^c^

Results in SSc patients (n = 38) versus controls (n = 26). Pearson correlation coefficient is given. ^a^*P *< 0.05, ^b^*P *< 0.01, and ^c^*P *< 0.001.

### Cytokine and chemokine levels correlated with LFTs and HR-CT scores for lung fibrosis in SSc

As shown in Table 2, several cytokines were increased in SSc-ILD patients when compared with ILD-negative SSc patients. The highest upregulated cytokine was CCL2; that was three-to fourfold increased when compared with healthy donors or ILD-negative SSc patients. Other cytokines such as IL-4, IL-6, IL-7, and IL-8 were two- to threefold upregulated in ILD-positive SSc patients compared with ILD-negative SSc patients.

In SSc patients, negative correlations were found between the predicted DLCO levels and the BALF concentrations of IL-2, IL-4, IL-8, and CCL2 (Table 4). Predicted FVC values also correlated negatively with the BALF IL-4 cytokine levels, IL-8, and CCL2 levels. We also found weak but significant correlations between the predicted TLC values and the BALF IL-4, IL-8, and CCL2 concentrations. No correlations were noted between IL-6/IL-7 concentrations and lung-function parameters.

**Table 4:** Linear correlation of cytokine levels and lung-function parameters as well as thoracic HR-CT scores

		IL-2	IL-4	IL-7	IL-8	CCL2
DLCO-SB (%)	Pearson *r*	-0.390	-0.536	-0.116	-0.409	-0.442
	*P *value	0.025	0.0013	0.492	0.012	0.006
FVC (%)	Pearson *r*		-0.503	-0.195	-0.394	-0.441
	*P *value		0.0028	0.248	0.016	0.006
TLC (%)	Pearson *r*		-0.362	-0.081	-0.331	-0.412
	*P *value		0.04	0.63	0.04	0.01
HR-CT score	Pearson *r*			-0.021		0.362
	*P *value			0.903		0.03

The predicted DLCO-SB, FVC, and TLC values are given in percentages. All significant correlations are shown.

We had 36 HR-CT scores derived from 30 SSc patients at the time of BAL. When comparing the HR-CT scores with the cytokine levels, a weak correlation was seen between CCL2 levels and the HR-CT scores (Table 4). Patients with an HR-CT score of 20 or greater had higher CCL2 levels when compared with patients with fewer fibrotic changes (*P *= 0.04, data not shown). No other cytokine concentrations were found to be related to HR-CT scores.

### BALF cytokine levels predict deterioration of lung fibrosis

**Figure 2:**
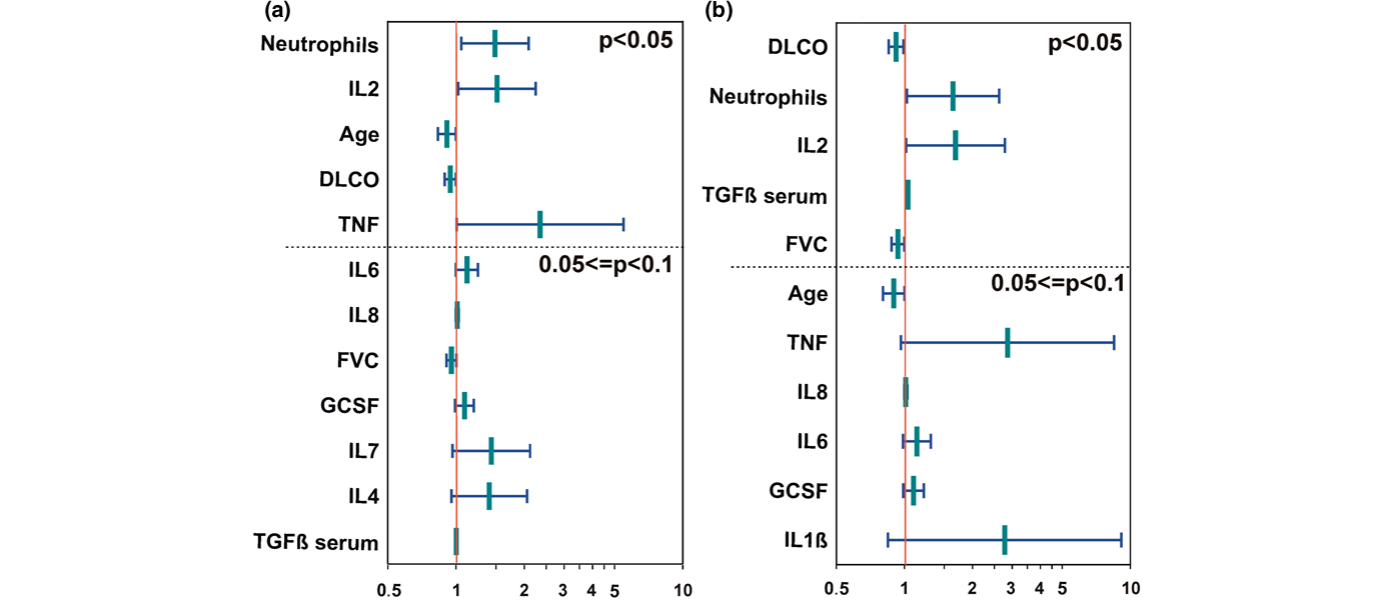
Odds ratios with confidence interval for **(a)** progressive versus stable interstitial lung disease (ILD) and **(b)** end-stage versus stable ILD from univariate logistic regression analysis. Illustrated are all parameters with *P *< 0.1, sorted by descending significance. The dashed line divides into significant parameters and parameters showing a trend. The y axis is log-transformed.

For 29 SSc patients providing 35 BAL samples (all multiple samples were from patients with end-stage ILD), follow-up investigations of ILD were available (29 HR-CT scans at a mean period of 58 weeks after BAL and 27 comparable lung function follow-up investigations at a mean period of 49 weeks after BAL). Furthermore, patients were evaluated for end-stage ILD for a mean period of 38 months after BAL. Of the 10 patients with progressive disease, 6 patients developed end-stage ILD. As shown in Table 5, high BALF cytokine concentrations of several cytokines, such as IL-2, IL-6, IL-8, and TNF-α, were associated with progressive or end-stage ILD. IL-7 levels showed a trend to be predictive for progressive and end-stage diseases compared with constant controls (*P *= 0.07 and *P *= 0.09, respectively). Additionally, neutrophilic alveolitis, reduced DLCO and FVC values, as well as young age were found to be more frequent in patients with progressive disease. In contrast, higher HR-CT scores and mRSS levels at the time of BAL were not associated with progressive lung disease (Table 5). By univariate analysis, predictors for progressive ILD were young age, low predicted DCLO levels, high IL-2 and TNF-α levels (*P *< 0.05), and a high percentage of neutrophils (Figure 2a). The latter was the best predictor for progressive ILD (p = 0.023). Predictors for end-stage ILD were again low predicted DLCO and FVC levels, high IL-2 levels, and a high percentage of neutrophils (Figure 2b, *P *< 0.05). All potential predictors identified by univariate analysis (*P *< 0.1) were subsequently tested with multivariate analysis. The stepwise forward- and backward-selection methods revealed different combined indexes, presumably caused by the many parameters tested in a relative small group of patients. For progressive disease, young age and neutrophilic alveolitis were selected in both modes, either combined with FVC or with IL-2, TNF-α, and IL-7. For the small group of end-stage ILD patients, neutrophilic alveolitis together with IL-1 and FVC revealed a predictive value. When only BALF cytokines were studied, high levels of IL-2 and TNF-α predicted progressive/end-stage ILD.

**Table 5:** Cytokine and chemokine concentrations from 35 SSc BALF samples related to the clinical characteristics and progression of ILD

Parameter	All BALF samples (n = 35)	Samples from stable ILD (n = 19)	Samples from progressive ILD (n = 16)	Samples from end-stage ILD (n = 12)
G-CSF	28.2 (1.4–191)	23.9 (1.4–45)	34.1 (20.2–191)^a^	32.6 (20.2–50.6)^a^
IL-1	0.9 (0–17.2)	0.5 (0–2.8)	1.3 (0.3–17.2)^b^	1.3 (0.3–17.2)^a^
IL-2	3.6 (0–24.9)	0.9 (0–7.6)	5.7 (0.9–24.9)^a^	5.7 (2.8–24.9)^b^
IL-4	0.4 (0–22.9)	0 (0–4.6)	3.5 (0–22.9)^b^	5.2 (0–22.9)^a^
IL-6	15.8 (1.7–104.6)	13.1 (6.2–30.7)	21.4 (1.7–104.6)^b^	21.4 (1.7–104.6)^b^
IL-7	4.43 (0–11.7)	3.5 (0–6.1)	4.88 (1.24–11.66)	4.88 (1.95–11.66)
IL-8	71.1 (5.9–794.5)	43.7 (5.9–209.3)	105.5 (22.4–794.5)^b^	124.2 (53.8–94.5)^b^
CCL2	47.1 (0–19,248)	44.6 (0–19,248)	92.2 (14.1–2,000.5)	139.4 (26.7–2,000)^a^
CCL4	45.5 (4.2–119.9)	39.5 (4.2–111.3)	53.9 (24.6–119.9)	53.9 (35–107.1)
TNF-α	0.9 (0–8.1)	0.3 (0–3.6)	1.7 (0–8.1)^b^	1.9 (0–8.1)^b^
Immunosuppressive therapy at the time point of BAL (during follow-up)				
CTX/MMF	8 (23)	1 (9)	8 (16)	7 (12)
AZA/others	8 (11)	6 (9)	2 (2)	1 (1)
Number	17 (3)	10 (3)	6 (0)	4 (0)
Patients	29	19	10	6
Age (y)	59 (37–72)	61 (37–72)	53 (38–63)^b^	49 (41–59)^b^
Disease duration (a)	3 (0.5–14)	3.5 (0.5–14)	3 (0.5–10)	3 (0.5–8)
S/Ex/NS (%)	3/7/19 (10/24/66)	1/3/15 (5/16/79)	2/4/4 (20/40/40)	1/4/1 (17/67/17)
Female	22 (76%)	15 (79%)	7 (70%)	4 (67%)
mRSS	13 (0–31)	14 (0–31)	10.5 (0–24)	11.5 (0–24)
HR-CT score	13.5 (0–27)	13.8 (0–27)	13 (0–27)	17 (0.7–27)
TLC (%)	81.2 (47.2–124)	81.2 (47.9–124)	79.4 (47.2–104)	65.4 (47.2–97.1)
FVC (%)	75.7 (29.3–108)	84.4 (29.3–108)	68.8 (43.3–92.7)^b^	62.2 (43.3–72.6)^b^
DLCO (%)	61.6 (20.4–108.3)	66.7 (32.9–108.3)	53.6 (20.4–68.4)^a^	36.6 (20–68.4)^b^
Neutrophils (%)	3 (0–49)	2 (0–9)	14.5 (3–49)^c^	16.5 (4–38)^c^
Macrophages	82 (39–98)	87.5 (39–98)	73 (41–93)^a^	74 (49–91)
Death	4 (14%)	1 (6%)	3 (30%)	3 (50%)

Progressive ILD was defined either by worsening of HR-CT (≥ 3) or by changes of the predicted FVC, DLCO-SB, or TLC values for ≥ 10% during a follow-up of 2.5 years. End-stage ILD was defined either by death or by the need for continuous oxygen supplementation. CTX = cyclophosphamide; MMF = mycophenolate mofetil; AZA = azathioprine; S = smoker; Ex = ex-smoker; N = non-smoker. Concentrations are given in picogram per milliliter. Median values and ranges are shown. *P *values of the Mann-Whitney test are given by ^a^*P *< 0.05, ^b^*P *< 0.01, ^c^*P *< 0.001.

## Discussion

In the present work, we studied cytokine and chemokine levels in BALF to identify key players in the disease process and potential therapeutic targets of SSc-related lung disease. Systemic sclerosis is a rare disease, and most studies analyzing BALF cytokines have included only few patients with SSc. Our analysis is one of the largest studies addressing soluble mediators in BALF associated with SScs. Furthermore, in contrast to other investigations that have used ultrafiltration or other methods to concentrate BALF cytokines and chemokines for ELISA testing [18-21], we used a highly sensitive Bioplex assay, allowing the detection of cytokines without any BALF manipulation that could influence the stability of cytokines. By using this technique, we observed abnormalities in a broad range of cytokines and chemokines, probably reflecting the complexity of the underlying disease processes present in SSc. Cytokines/chemokines produced by lymphocytes (for example, IL-4, IL-2) and monocytes/macrophages (CCL2, CCL4, TNF-α, IL-8, IL-6), as well as other cell types, were shown to be increased, indicating activation of different cell types in SSc. In controls with ILD due to other diseases, fewer abnormalities were observed; however; some cytokines/chemokines, such as CCL2 and CCL3, were increased in idiopathic ILD as well as in SSc-associated ILD, indicating an important, but nonspecific contribution of these chemokines in lung fibrosis. As tested here for SSc patients, CCL2 concentrations were found to be correlated with lung-function parameters and HR-CT score. The importance of CCL2 as a key mediator for lung fibrosis also is supported by data from animal models showing a reduction and prevention of bleomycin-induced lung fibrosis by anti-CCL2 monoclonal antibodies or by pharmacologic blockade, respectively [22,23]. CCL2 also mediates profibrotic effects in SSc through the release of IL-4 from T cells, and IL-4 also was found to correlate with lung-function parameters in our study [24]. Taken together, our data support the role of CCL2/3 as targets for future therapies.

Additional cytokines and chemokines, such as IL-8, also could be important, as IL-8 gene polymorphisms are associated with an increased risk of SSc [25]. IL-8 also is expressed by scleroderma fibroblasts and by alveolar macrophages [26-28], and increased IL-8 levels in BALF and serum of SSc patients have been described by others [18,29]. By reducing multivariate logistic regression analysis on BALF cytokines, high IL-8 levels also were predictive of a poor prognosis. IL-8 is a potent chemoattractant for neutrophils, and the correlation of IL-8 levels with LFTs suggests a possible role of this cytokine in ILD pathogenesis, as suggested by others [29]. Therefore, IL-8 could also serve as a potential therapeutic target.

In comparison with alveolitis due to other diseases, SSc-related alveolitis was characterized by higher levels of IL-7, suggesting disease-specific pathogenic processes. IL-7 was originally described as a potent proliferative stimulus of pro-B and pre-B cells from bone marrow [30] and as a promoter of the growth and expansion of mature effector T cells [31]. It is expressed by stromal medullar cells, epithelial cells, and macrophages [32] and exhibits both fibrotic and antifibrotic effects, probably underlined here by the missing correlations between IL-7 levels and LFTs or HR-CT scores detectable for other cytokines. IL-7 transgenic mice showed increased levels of the profibrotic cytokines IL-4 and IL-13 [33]. Here, higher IL-4 levels also were detected in SSc-associated alveolitis. The antifibrotic effect of IL-7 is reflected by an improvement of bleomycin-induced pulmonary fibrosis through IL-7 [34]. This effect could explain the better prognosis of SSc-associated alveolitis compared with that of idiopathic ILD [35].

However, as suggested by our study, the activation of T cells by IL-7 could be important for SSc-ILD. In line with this, and in addition to the clinical associations of IL-4, IL-2 concentrations correlate with predicted DLCO levels. Furthermore, high IL-2 levels together with high TNF-α levels were the best predictors for progressive/end-stage ILD. Increased levels of the IL-2 receptor are proposed as a marker of disease activity in SSc [36] and SSc-associated ILD [37]. In line with this, blockade of the IL-2 receptor activation ameliorated bleomycin lung fibrosis [38].

In another study, anti-CD3 therapy also diminished bleomycin-induced fibrosis [39]. Therefore, T-cell therapy could provide a useful target for further therapies.

Because most of the fibroblast characteristics obtained from SSc patients are reproduced in normal fibroblasts after stimulation with TGF-β1, TGF-β1 was stated as a key cytokine in SSc-associated fibrosis (reviewed in [40]). Here, BALF TGF-β1 levels were below the detection level. In contrast, serum TGF-β1 levels were detectable by using the same assay but did not provide any correlations with LFTs or HR-CT scores and revealed no predictive capacity (Figure 2). The low levels of potential profibrotic cytokines found in our study do not exclude autocrine or paracrine effects, as suggested by others to play a role in SSc [41]. However; several cytokines analyzed here and showing increased concentrations exhibit inhibitory effects on TGF-β1 expression (for example, IL-7 and TNF-α), indicating a contribution of TGF-β1-independent mechanisms in SSc-ILD proposed by others, such as autoantibodies, Th2 cytokines, growth factors, and several other cytokines/chemokines [42,43]. As detected with our first analysis including 17 different cytokines, profibrotic cytokines such as IL-13 or IL-17 also revealed very low or undetectable BALF concentrations and no differences from controls or a relation to ILD. Levels of IL-17 correlated with BAL lymphocytosis but not with clinical parameters in SSc (data not shown). Because BAL lymphocytosis is associated with stable lung function over time [44], IL-17 does not seem to play a major role in SSc-ILD. Despite the possible important role of lymphocyte activation and cytokine release in SSc-ILD, accumulation of lymphocytes in BAL was not predictive of disease progression.

Instead, and supporting studies from several groups, we found neutrophilic alveolitis as one of the strongest predictors for progressive disease. The role of neutrophilic alveolitis and BAL analysis as predictors of progressive disease was recently discussed [45]. Several observational studies, including a total of 190 SSc patients, indicated that the presence of BAL alveolitis, and especially of neutrophilic alveolitis, was associated with deterioration of lung-function tests in patients that did not receive immunosuppressive treatments (summarized in [45]).

In contrast, a recent analysis of 66 placebo-treated patients from the Scleroderma Lung Study did not show any relation between the presence of baseline BAL granulocytosis and changes in lung function [4]. As recently outlined, a previous study was not sufficiently powered to allow subgroup stratification [45]. Furthermore, undetected infections, technical issues such as the instilled volume of saline, the site from which BAL was performed, different cut-offs used to define alveolitis, or comorbidity such as reflux can influence BAL cellularity (summarized in [45]). This could lead to different results, as reported in other studies [3,4]. Here, in this single-center study, we used a standardized procedure, and we have adjusted the cytokine concentrations for BAL recovery. By this procedure, TNF-α, a cytokine with the capacity to increase the migration of neutrophils, was found to be one of the best predictors for poor prognosis. This cytokine was found to correlate with the absolute number and frequency of BAL neutrophils (Table 3). In line with this, our study supports the predictive value of neutrophilic alveolitis, which could be different from granulocytic alveolitis, because eosinophils did not reveal any predictive capacity in SSc. However, our study is not powered to provide conclusive information about the value of BAL to predict disease progress. Nevertheless, cytokines found to be predictive, even in our small patient sample, have promise of a high prognostic potential. Their role in a multivariate setting in addition, to known prognostic factors, must be assured with higher case numbers. Further studies are needed to address this question.

Major limitations of the study are the fact that the majority of patients received immunosuppressive therapies at the time of BAL. Therefore, it cannot be excluded that immunosuppressants could influence BALF cytokines and, subsequently, cytologic and clinical correlations. However, in the few patients investigated twice or thrice, no significant BAL changes were observed despite immunosuppressive therapies. Another limitation of the study is the low number of patients and the heterogeneity of the control group.

In conclusion, we identified several abnormalities in the cytokine and chemokine patterns in BALF of SSc patients, suggesting an important role of these mediators in the pathogenesis of ILD. According to our results, CCL2, IL-7, and probably IL-8 and IL-4 appear to be the most-promising candidates for a targeted therapy in SSc-associated ILD. Furthermore, T-cell targeted therapy could be a promising therapeutic intervention. The data also suggest the usefulness of BALF analyses as an early predictor of progression of SSc-related ILD.

## Conclusions

High BALF cytokine and chemokine levels are associated with severe ILD in SSc and are associated with deterioration of ILD. Cytokines and chemokines could have a role in the disease pathogenesis of ILD. Analyses of BALF chemokine and cytokine levels can probably provide therapeutic targets in SSc-associated ILD.

## Abbreviations

ACR: American Congress of Rheumatology; BAL: bronchoalveolar lavage; BALF: bronchoalveolar lavage fluid; CT: computed tomography; DLCO: predicted diffusion capacity; DNSS: German Network (Deutsches Netzwerk) of Systemic Scleroderma (DNSS); dSSc: diffuse SSc; ELISA: enzyme-linked immunosorbent assay; EUSTAR: European Scleroderma Trial and Research network; FVC: predicted forced vital capacity; HR-CT: high-resolution computed tomography scan (HRCT); HU: Hounsfield units; ILD: interstitial lung disease; LFP: lung-function parameter; lSSc: limited SSc; MRSS: modified Rodnan Skin Score; SD: standard deviation; SSc: systemic sclerosis; TLC: total lung capacity.

## Competing interests

The authors declare that they have no competing interests.

## Authors' contributions

K Schmidt and L Martinez-Gomboa L performed the detection of cytokine concentrations; K Schmidt also performed some statistical analyses and generated the graphs and tables. S Meier analyzed the HR-CT scans and derived the HR-CT score, together with the pulmonologists C Witt and L Hanitsch. M Becker M wrote and corrected the manuscript. D Huscher provided statistical support and conducted the logistic regression analyses. C Meisel supervised BAL cell differentiations and provided these data for further analyses. G Burmester discussed the data with the last author and made intellectual contributions. G Riemekasten, as the last and responsible author, initiated this study and controlled the work. She initiated the study, collected the patient data, assessed the patients, and wrote and reviewed the manuscript.
